# Reducing Employee Injury Rates with a Hospital-wide Employee Safety Program

**DOI:** 10.1097/pq9.0000000000000387

**Published:** 2021-02-12

**Authors:** Alia Fink, Kathryn Merkeley, Charika Tolliver, Raven McLeese, Janice J. Mason, Nikolas Mantasas, Jenhao Jacob Cheng, Reneè Roberts-Turner, Lisbeth Fahey, Martha Parra, Linda Talley, Rebecca Cady, Rahul K. Shah

**Affiliations:** From the * Children’s National Hospital, Washington, D.C.; † The George Washington University School of Medicine and Health Sciences, Washington, D.C.; ‡ Alia Fink and Katheryn Merkeley are co-first authors.

## Abstract

**Introduction:**

Despite the well-known dangers of working in the healthcare industry, healthcare organizations have historically accepted workplace injuries as business as usual. In 2017, Children’s National Hospital began our Employee and Staff Safety program to drive down the employee injury rate and address this disturbing industry trend.

**Methods:**

With guidance and support from executive leadership, we created an Employee and Staff Safety program that aligned employee safety work with existing patient safety and quality improvement efforts. Team leads collected and analyzed baseline employee injury data and identified areas of highest injuries. Dedicated subcommittees focused on five specific areas: slips, trips, and falls; sharps injuries; blood and body fluid exposures; verbal and physical violence; and overexertion injuries. Subcommittees established aims, identified key drivers, and brainstormed interventions for tests of change.

**Results:**

Because the inception of the Employee and Staff Safety program, Children’s National has seen significant reductions in our Days Away Restricted or Transfer (DART) rate. The DART rate shows a sustained 37% reduction since the baseline period of FY16–FY17 (1.48 injuries/200,000 h worked to 0.93 injuries/200,000 h worked). The regression trend shows a significant decrease (38.3%) in DART injuries, from 1.544 to 0.952 over 56 months; *P* = 0.016.

**Conclusions:**

Active leadership support and analyzing data on specific employee harm areas coupled with targeted interventions, helped improve Children’s National’s DART rate. The Employee and Staff Safety program’s success in utilizing patient safety and quality improvement tools creates a generalizable framework for other hospitals to advance their high-reliability journey.

## INTRODUCTION

Healthcare is a risky business, totaling more injuries than manufacturing and construction.^1^ Serious, nonfatal workplace injuries account for $99.4 million/wk in workers’ compensation costs and subsequently lead to burnout, high turnover, decreased productivity, poor patient outcomes, and contribute to healthcare workforce shortages.^2–4^ Because the formation of the Occupational Safety and Health Administration (OSHA) in 1971, workplace injuries and deaths reduced from 10.9 incidents per 200,000 hours worked in 1972 to 2.8 per 200,000 hours worked in 2017.^1^ Despite the inherent risks of working in healthcare, injuries have historically been accepted as business as usual, a cost of providing care.

After the release of *To Err Is Human* in 1999, patient safety became the central focus of healthcare.^5^ Organizations adopted high-reliability principles widely used in risky industries such as aviation and nuclear power to reduce patient harm. High-reliability industries operate high risk, complex systems for long periods with few serious incidents by embedding 5 principles into daily operations: preoccupation with failure, reluctance to simplify, sensitivity to operations, commitment to resilience, and deference to expertise.^6^ Similar to aviation and nuclear power, the stakes are high for healthcare organizations, and significant incidents have serious, often highly visible consequences.

With the primary focus admittedly on *patient* safety, organizations did not allot the same resources and attention to employee safety. Historically, employee safety was the domain of Occupational Health, Risk, and Worker’s Compensation. Children’s National Hospital took a similar approach until 2017, when our President and Chief Executive Officer engaged leadership to examine the positive relationship between workplace safety and patient outcomes.^7^ Leadership reviewed existing evidence on effective employee safety programs for specific injury types. It noted a lack of studies on comprehensive safety programs aimed at reducing overall employee harm.^8–10^ Baseline data for Children’s National demonstrated a Days Away Restricted or Transferred (DART) rate higher than the national benchmark. DART, an OSHA safety metric, indicates how many employees are injured or ill, requiring them to miss work, be on restricted duties, or transfer to another job.^1^ In November 2017, Children’s National’s DART rate was 1.48 injuries per 200,000 hours worked compared to a benchmark of 1.15.^11^ Executive leadership encouraged the organization to launch a centralized, hospital-wide Employee and Staff Safety (ESS) program to create a safer work environment, which resulted in an aim to reduce the DART rate of 1.48 by 20% by December 2018 and sustain for 1 year.

## METHODS

This project is exempt from Institutional Review Board oversight because it is a quality improvement initiative that does not constitute human subjects research. Children’s National, located in Washington, D.C., is a 323-bed free-standing academic pediatric hospital with approximately 7,000 employees. In the early 2000s, Children’s National embarked on a high-reliability journey for safety and quality. The organization established the structure for a patient safety program by committing resources and adopting proven safety and performance improvement methodologies. Leadership charged those same resources and expertise with establishing a comprehensive patient and employee safety program.

**Fig. 1.**
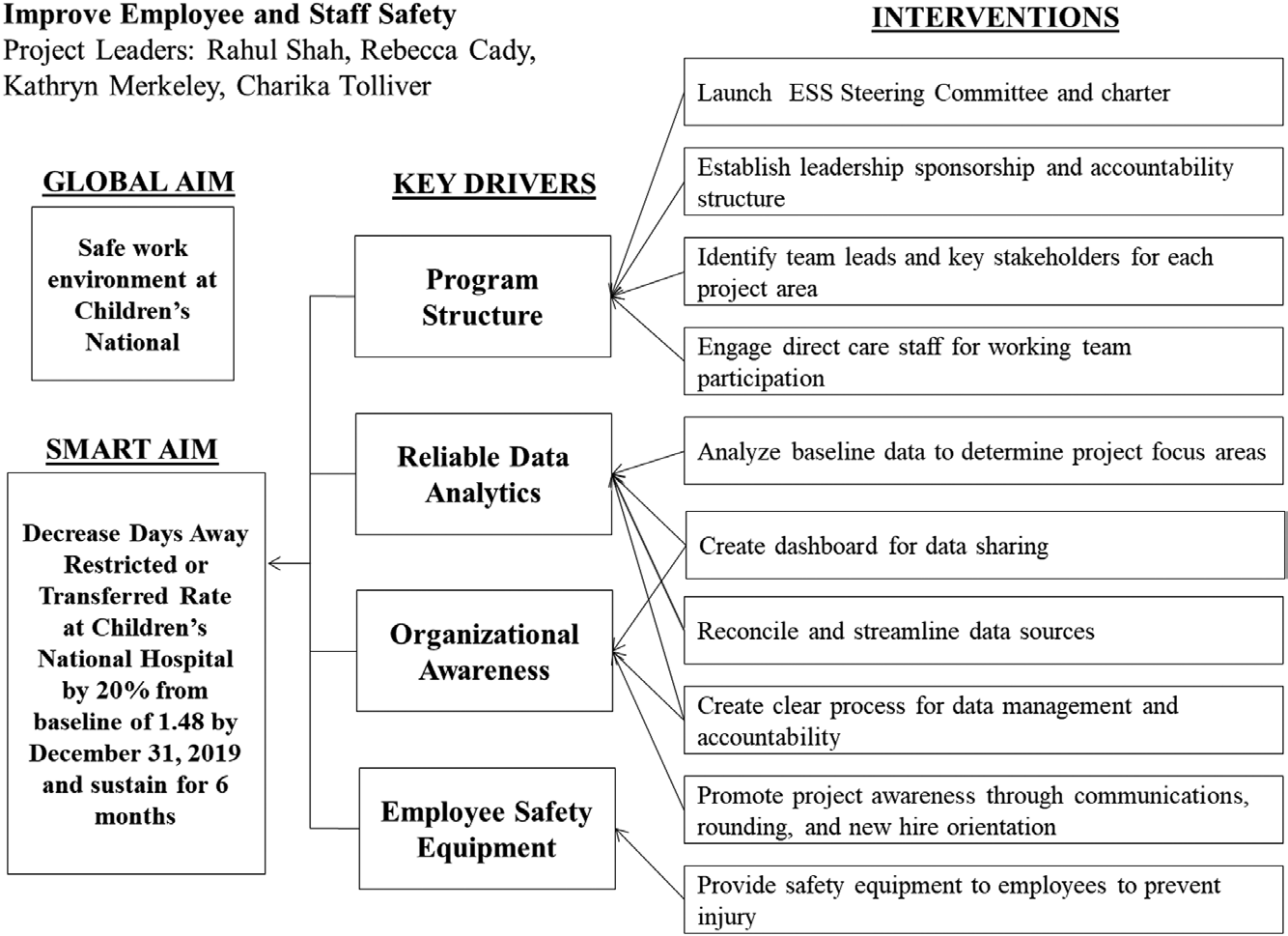
Key driver diagram. The ESS Steering Committee used the key driver diagram for project management and communication with stakeholders.

Launched in November 2017, the ESS Steering Committee took accountability for advancing Children’s National’s high-reliability culture by improving safety and reducing harm to employees. The ESS Steering Committee focused on creating structures and processes necessary for reducing employee harm. The committee identified the key drivers as program structure, reliable data analytics, organizational awareness, and safety equipment (Fig. 1).

### Program Structure

The ESS Steering Committee, co-chaired by the Vice-President, Chief Quality and Safety Officer and the Vice-President, Chief Risk Officer, is a multidisciplinary group with representatives from Risk Management, Patient Safety, Workers’ Compensation, Nursing, Security, Environmental Services, Occupational Health, Human Resources, and Performance Improvement. The membership provided a diverse range of operational perspectives and expertise. Utilizing the high-reliability principle of deference to expertise, members represented subject matter experts in patient and employee safety and had the oversight to address common employee injury types. The ESS Steering Committee reports to the executive-level Quality and Safety Steering Committee, charged with advancing care outcomes, safety, and service excellence. The ESS Steering Committee is ultimately accountable to the board-level Quality and Safety Council, dedicated to ensuring optimal outcomes through quality and safety integration.

Review of baseline data from safety event reports, Occupational Health records, and Worker’s Compensation events highlighted employee injury focus areas based on the frequency of events and staff most vulnerable to harm. Focus areas include slips, trips, and falls; blood and body fluid exposures; sharps injuries, workplace violence, and overexertion injuries. Operational definitions (Table 1) for each focus area, based on established OSHA definitions and Children’s National policies, ensured a standard nomenclature and evaluation criteria for employee injury events.

**Table 1.** Operational Definitions

Project	Operational Definition
Sharps injuries	Break in the skin related to use, handling, disposal of needles, scalpels, razors, or sharp instrument objects
Blood and body fluid exposures	Contact with blood or other potentially infectious materials to eye, mouth, other mucous membrane, non-intact skin, or parenteral
Workplace violence: employee vs. employee, patient vs. employee, and caregiver/visitor vs. employee	Verbal violence: name calling, obscene language, or other abuse behavior, and intimidation through direct or veiled threats Physical violence: throwing objects, physically touching, or physically intimidating
Slips, trips, and falls	Slip: lack of traction between footwear and walking surface Trip: lower extremity movement impeded Fall: total loss of balance from sane level or lower level
Overexertion	Acute or chronic musculoskeletal injury from outside sources, repetitive motions, and other exertions such as: lifting/moving, twisting/turning, reaching, pulling/pushing, typing/texting, sitting/standing, or kneeling resulting in days away, restricted, or transferred

The ESS Steering Committee named team leads for each focus area based on data trends, subject matter expertise, and oversight necessary to drive change. Subcommittee make-up deliberately included subject matter experts and direct-care staff. Patient Safety and Performance Improvement staff served as consultants for the subcommittees and provided expertise in improvement methodologies.

Recognizing the need for a cohesive approach to reducing workplace violence, the ESS Steering Committee and workplace violence team-lead worked in conjunction with the Disruptive Patient Taskforce (DPT). Launched in 2016, the DPT targeted staff competency, facility infrastructure, and the clinical care model for reducing disruptive patient events. The ESS Steering Committee focuses on employee-only safety events, whereas the DPT focuses on all disruptive patient events that may or may not lead to employee events.

Concurrent with the inception of the ESS program, Children’s National joined the Children’s Hospitals’ Solutions for Patient Safety (SPS) Network Overexertion Pioneer Cohort to reduce employee overexertion injuries. The SPS cohort focused on testing injury prevention interventions to decrease employee injuries from lifting and moving patients. Alignment with the SPS cohort allowed for shared learning and problem solving with other pediatric hospitals.

Team leads and subcommittees created key drivers through baseline data analysis, staff feedback, literature reviews, and best practices. Themes such as environment, training, process and technique, awareness, and safety equipment emerged. Subcommittees identified interventions and pilot areas through Pareto charts, fishbone diagrams, risk assessments, and facilitated feedback sessions. Plan-Do-Study-Act cycles facilitated systematic implementation and evaluation of interventions. Team leads set project timelines and continuously reassessed data and outcomes to determine interventions for spread to other areas and guide future work.

### Reliable Data Analytics

Clear operational definitions streamlined data analysis and management. Employee injury data came from safety event reports, Occupational Health records, and Worker’s Compensation events. Clear and specific criteria for evaluating and classifying events helped create a reliable system for tracking and trending employee harm. For example, an escalating parent event may be entered as verbal violence in the safety event reporting system, but not meet the definition of workplace violence as there was no direct threat toward an employee. Before inclusion in the data, the team leads carefully review and evaluate events against the operational definitions.

**Fig. 2.**
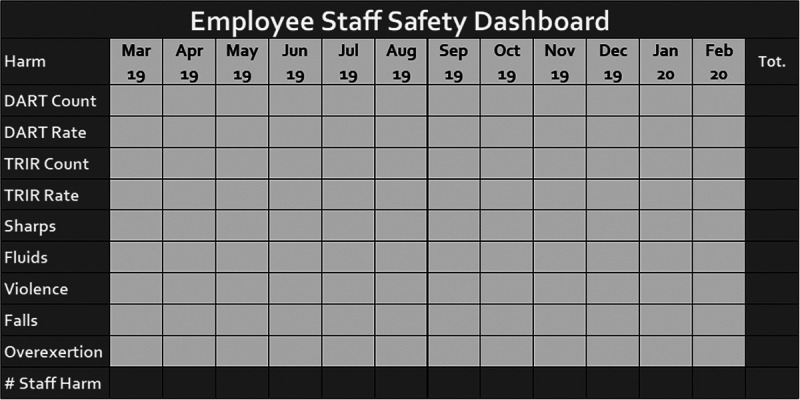
Employee Staff Safety Dashboard. The ESS Dashboard displays employee safety metrics on a rolling 12-month basis.

The ESS Steering Committee created the ESS Dashboard for tracking employee injuries (Fig. 2). The dashboard intentionally mirrors our Zero Harm Index, which displays patient harm data. A Preventable Harm Index was shown by Brilli et al^12^ to be a catalyst for driving sustained improvements in hospital-acquired conditions.^13^ The ESS Dashboard tracks monthly DART and Total Recordable Incident Rate information and counts for each focus area. Data displayed over a rolling 12-month period provides situational awareness and promotes timely response to potential risks. Regular trending of dashboard data supports preoccupation with failure by identifying areas for the ESS Steering Committee, other organizational quality and safety committees, and the hospital board to monitor.

The ESS Dashboard centralized outcome tracking for the ESS Steering Committee and subcommittees. The DART rate was the primary outcome measure, and subcommittees established secondary outcome and process measures based on individual projects. For example, the blood and body fluid exposure subcommittee tracked monthly personal protective equipment (PPE) compliance, and the Workplace Violence subcommittee tracked the number of visitor violence event reports and the number of staff who attended Crisis Prevention Intervention de-escalation training.^14^ Balancing measures included safety climate and working condition scores on the 2018 Safety Attitudes Questionnaire. Children’s National administers a safety culture survey every 18 months to 2 years. These scores reflect staff perception of safety and working conditions during the transition from a patient-focused program to a comprehensive patient and employee-focused safety program. The ESS Steering Committee chose this metric as emerging literature shows a positive relationship between safety climate and employee safety climate and recommends the convergence of patient and ESS programs.^15^

### Organizational Awareness

Promoting engagement from direct-care staff and leaders involved raising organizational awareness about the program and commitment to reducing employee harm. “Your Safety Matters Too,” a campaign launched in 2018 to educate staff about the project, encouraged employee injury reporting and speaking up when staff identify a risk. ESS Steering Committee members emphasized the organizational commitment to patient *and* employee safety as equal priorities. Team leads and ESS Steering Committee members rounded with staff regularly to raise awareness. Additionally, the Daily Check-In safety briefing incorporated reports on employee injury risks or events to increase situational awareness and escalate concerns.^16^ The inclusion of reporting employee safety in the Daily Check-In supported our high-reliability focus on sensitivity to operations and employee safety as an organizational priority.

ESS content integration into new hire training, a 1-day orientation dedicated to safety and service for all new employees, ensured staff coming into the organization prioritized patient *and* employee safety. New hires learned about the focus areas for employee safety and how to respond to and report employee injuries.

### Safety Equipment

Data analysis and direct-care feedback identified safety equipment as a key driver for each focus area. Specific equipment needs for each focus area varied; access to appropriate safety equipment emerged as a common risk. The ESS Steering Committee, team leads, and subcommittees carefully evaluated equipment options, with input from direct-care staff, to ensure optimal equipment purchases and thorough implementation strategies. Safety equipment examples for each focus area include:

Slips, trips, and falls: Purchased additional wet floor signs and placed them in high traffic areas.

Sharps injuries: Collaborated with materials management to trial, purchase, and implement needleless transfer devices for blood and medications.

Blood and body fluid exposures: Identified appropriate PPE for high-risk procedures and optimal placement on units to support workflow.

Overexertion injuries: Obtained mechanical lifts and friction-reducing slider sheets for inpatient units.

Workplace violence: In conjunction with the DPT, Security personnel used Kevlar sleeves to prevent skin injuries in high-risk areas such as the Emergency Department.

The subcommittees continuously evaluate equipment needs, expand purchasing, and spread to new areas.

## RESULTS

### Outcome Measures

**Fig. 3.**
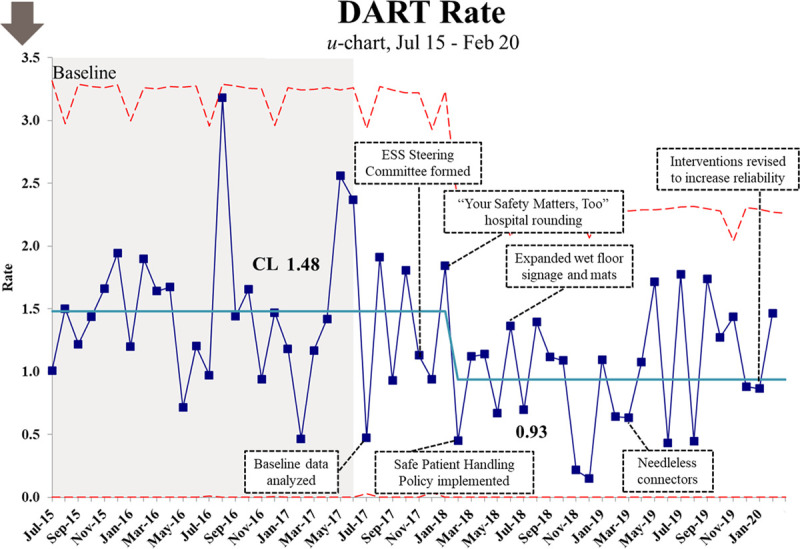
Dart Rate u-Chart. Statistical process control chart with annotations showing DART rate data over the reporting period.

Monthly injury rates for DART, and each project focus area is calculated using the number of injuries per 200,000 hours worked. Children’s National had a 37% reduction in the DART rate from the baseline period of FY16/FY17, from 1.48 to 0.93 (Fig. 3). The regression trend demonstrates a 38.3% decrease in DART from 1.544 to 0.952 over 56 months with a slope of −0.011 (95% confidence interval [CI]: −0.020, −0.002; *P* = 0.016). The total DART injury rate decreased by 40.5% from 1.541 in FY17 to 0.916 in FY 2019 for a difference of −0.624 (95% CI: −1.037, −0.211; *P* = 0.003).

Though the DART rate improved, results from individual metrics were mixed. Table 2 shows percent changes between FY17 to FY19 for DART injuries and each project focus area. Total slips, trips, and falls decreased by 36.7% in stepwise increments from 1.669 in FY17 to 1.057 in FY19 for a difference of −0.612 (95% CI: −1.047, −0.177; *P* = 0.006). Between FY18 and FY19, overexertion injuries related to lifting and moving patients reduced by 58.6% from 0.085 to 0.035 for a difference of −0.050 (95% CI: −0.139, 0.039; *P* = 0.309), whereas sharps injuries decreased by 11.2% from 1.210 to 1.075 for a difference of −0.135 (95% CI: −0.525, 0.255; *P* = 0.499), both reductions are not statistically significant. Blood and body fluids exposures almost remained the same between FY17 and FY19, slightly increased by 0.6% from 0.770 to 0.775 for a difference of 0.005 (95% CI: −0.322, 0.332; *P* = 0.977). Violence increased by 11.6% during the reporting period from 3.283 to 3.665, but such an increase is not statistically significant, with a difference of 0.382 (95% CI: −0.310, 1.074; *P* = 0.2861). The significance tests are based on Poisson distribution because the data are the counts of rarely occurring safety events.

**Table 2.** FY 2017–FY 2018 Changes

Project Focus Area	F17 Rate	F18 Rate	% Change	Difference	Lower Limit	Upper Limit	*P*
Total number DART cases	1.541	1.125	−27.0	−0.416	−0.842	0.011	0.056
Blood and body fluids exposures	0.770	0.733	−4.9	−0.037	−0.357	0.282	0.819
Overexertion	0.110	0.239	116.8	0.129	−0.024	0.281	0.109
Overexertion from lifting and moving patients	0.073	0.085	16.2	0.012	−0.092	0.116	0.836
Sharps	0.807	1.210	50.0	0.403	0.034	0.772	0.033
Slips, trips, falls	1.669	1.415	−15.2	−0.254	−0.713	0.204	0.277
Workplace violence	3.283	3.051	−7.1	−0.232	−0.889	0.425	0.489
FY 2018–FY 2019 Changes							
Project Focus Area	F18 Rate	F19 Rate	% Change	Difference	Lower Limit	Upper Limit	*P*
Total number DART cases	1.125	0.916	−18.6	−0.209	−0.577	0.160	0.270
Blood and body fluids exposures	0.733	0.775	5.8	0.042	−0.275	0.359	0.794
Overexertion	0.239	0.282	18.1	0.043	−0.143	0.230	0.654
Overexertion from lifting and moving patients	0.085	0.035	−58.6	−0.050	−0.139	0.039	0.309
Sharps	1.210	1.075	−11.2	−0.135	−0.525	0.255	0.499
Slips, trips, falls	1.415	1.057	−25.3	−0.357	−0.763	0.048	0.085
Workplace violence	3.051	3.665	20.1	0.614	−0.055	1.283	0.072
FY 2017–FY 2019 Changes							
Project Focus Area	F17 Rate	F19 Rate	% Change	Difference	Lower Limit	Upper Limit	*P*
Total number DART Cases	1.541	0.916	−40.5	−0.624	−1.037	−0.211	0.003
Blood and body fluids exposures	0.770	0.775	0.6	0.005	−0.322	0.332	0.977
Overexertion	0.110	0.282	156.2	0.172	0.008	0.336	0.043
Overexertion from lifting and moving patients	0.073	0.035	−52.0	−0.038	−0.125	0.049	0.425
Sharps	0.807	1.075	33.2	0.268	−0.092	0.628	0.147
Slips, trips, falls	1.669	1.057	−36.7	−0.612	−1.047	−0.177	0.006
Workplace violence	3.283	3.665	11.6	0.382	−0.310	1.074	0.286

### Process Measures

Team leads and subcommittees determined process measures to track the effectiveness of interventions at reducing harm. For example, the blood and body fluids subcommittee monitored PPE compliance, which averaged 90% for FY17–FY19. Following the “Your Safety Matters Too” campaign that focused on understanding employee safety risks through increased event reporting, visitor violence event reports rose 43% in FY19. By the end of FY19, over 1,000 staff had trained in Crisis Prevention Intervention strategies. For overexertion, the team tracked staff compliance using mechanical lift equipment, which demonstrated a 36% increase from a baseline of 61%–83% since 2017.

### Balancing Measures

The ESS Steering Committee considered the impact of aligning patient and employee safety programs on organizational safety culture survey scores. Survey scores reflect staff perceptions of safety as an organizational priority and how the workplace supports safe operations. A comparison of safety culture survey scores showed a 4.6% increase in safety climate from 69% in 2017 to 73.6% in 2018. Working conditions scores improved by 6.4% from 49% in 2017 to 55.4% in 2018.

## DISCUSSION

Employee injuries in healthcare were noted in the early 1990s, but the emerging patient safety movement overshadowed these risks.^5,17^ Although Children’s National worked to improve patient harm by building a robust patient safety program, employee safety remained independent of these efforts.^17^ Children’s National’s high-reliability journey focused on anticipating and responding to safety risks to reduce patient harm. Limiting the safety program focuses on patients eliminating a significant contributor to the overall safety and culture of the organization, employees.

Chassin and Loeb^13^ identified 3 interdependent elements that organizations must establish to become highly reliable: leadership commitment, the organizational culture of safety, and tools for process improvement.^18^ Children’s National incorporated these elements in the ESS program. Positive results in safety climate and working conditions scores demonstrate the effectiveness of establishing employee safety as an organizational priority and creating systems and processes to support safe operations. Inclusion of high-reliability principles and the Donebedian framework of structure, process, and outcome, already utilized in patient safety work, created alignment and streamlined processes for establishing an overarching safety program. This alignment helped with employee “buy-in” and accelerated improvement efforts.

The ESS Steering Committee and subcommittees integrated high-reliability principles and the Donabedian framework into the program design.^19,20^ Structures allotted to reducing harm included the ESS Steering Committee, subcommittees, ESS Dashboard, and leadership briefings. We tracked measurable employee injury outcomes to work toward the global aim of a safe work environment at Children’s National.

The ESS Dashboard remains an influential structure for supporting employee safety in our organization. Emulating the previously mentioned Zero Harm Index, the ESS Dashboard is a familiar visual tool for promoting transparency, improving outcomes, and shifting from a patient safety focus to an overall safety program. It is a visual reminder for executive leadership and the Board of Directors to quickly gauge where the organization stands regarding ESS.

The ESS Steering Committee and subcommittees used a systematic approach for harm reduction, but progress was not linear. Subcommittees faced challenges such as direct-care staff reluctance to change practice and organizational culture that accepted workplace injuries as a part of the job. Subcommittee members demonstrated sensitivity to operations and deference to expertise by partnering with direct-care staff to understand workflow, identify operational needs, and implement sustainable interventions. Team leads demonstrated a commitment to resilience by responding quickly to events and working with staff to understand and address the associated risks.

At first, subcommittees could easily address the major causes of employee injuries. However, after teams implemented interventions to prevent the most common injury causes, it became more challenging to identify interventions for more complex causes. The ESS Steering Committee adopted the high-reliability principle of reluctance to simplify through event analysis and willingness to examine each event’s details. Focus shifted from the number of events to a systematic, multidisciplinary review of employee injury events. The analyses engaged a wide range of expertise in solving complex problems. Sustaining progress and revitalizing the work is a continuous process that requires an ongoing review of current structures and processes. Subcommittees update key drivers and project aims twice a year to adapt to the changing environment and maintain a sense of urgency.

Limitations of our work include the use of DART as a proxy measure for employee safety in the workplace; as this is the industry standard, it was utilized. Furthermore, DART only measures more significant injuries and does not account for less severe injuries. With the lack of an appropriate metric for measuring employee safety, using DART data, a metric that healthcare organizations already report to OSHA, is the only objective measure available. As this was a new initiative, we worked diligently to capture as many events as possible. We believe that increases in metrics such as workplace violence and overexertion injuries are attributed to increased organizational awareness and reporting.

Children’s National’s ESS program has been iterative and embedded in continuous improvement. Our program incorporates best practices derived by high-reliability industries and leaders in healthcare worker safety. Teams used data to target work and leverage existing resources to apply the right expertise to each area. Subcommittees continue to analyze, review, and revise key drivers and interventions to sustain improvement. The next steps include expanding the ESS Steering Committee to include additional stakeholders and exploring innovations to reduce workplace violence. The structure and processes described are generalizable to other hospitals that wish to integrate patient and employee safety programs. Alignment of existing resources with employee safety efforts makes the approach to reducing employee harm broadly applicable and attainable.

## DISCLOSURE

The authors have no financial interest to declare in relation to the content of this article.
